# Association of Evening Shifts, Night Shifts, and Consistent Overtime Exceeding 10 Hours per Day with Sudden Cardiac Arrest: A Case–Control Study

**DOI:** 10.3390/jcm13185393

**Published:** 2024-09-12

**Authors:** Seung Won Ha, Seung Mok Ryoo, Sang-Min Kim, June-Sung Kim, Hyojeong Kwon, Hanna Park, Dongju Kim, Won Young Kim

**Affiliations:** Department of Emergency Medicine, Asan Medical Center, University of Ulsan College of Medicine, Seoul 05505, Republic of Korea

**Keywords:** cardiac arrest, risk factor, occupations, case–control study

## Abstract

**Background:** We investigated the relationship between employment status, work patterns, and sudden cardiac arrest (SCA). **Methods:** This was a case–control study from September 2017 through December 2022 involving 17 emergency departments and 9 public health centers. The cases included patients aged 20–79 years with SCA, excluding those with traumatic arrest, terminal illness, pregnancy, unreliable information, or a “Do Not Resuscitate” order. Controls were selected from various health screening centers in Korea. All participants completed structured questionnaires. Propensity score matching was used to ensure comparability by age, sex, and socioeconomic status. **Results:** Of the 1536 patients enrolled, 116 from the case group were excluded due to missing employment data, leaving 1420 cases and 2304 controls for analysis. Employment was reported by 47.5% of cases and 59.4% of controls. There was no significant difference in the proportion of sole proprietors (20.6% vs. 22.5%, *p* = 0.39). The case group had a higher proportion of employers (13.2% vs. 6.5%, *p* < 0.001) and fewer employees (63.3% vs. 69.1%, *p* = 0.02). Professional roles were more common among controls (23.6% vs. 31.6%, *p* < 0.001), while labor-intensive jobs were more frequent in cases (27.7% vs. 17.8%, *p* < 0.001). The case group had more evening and night shifts (odds ratio [OR]: 1.04, 95% confidence interval [CI]: 1.01–1.06; OR: 1.05, 95% CI: 1.01–1.09) and longer workdays (OR: 1.06, 95% CI: 1.03–1.08). **Conclusions:** SCA patients were more likely to work evening and night shifts and have longer workdays, often exceeding 10 h.

## 1. Introduction

Sudden cardiac arrest (SCA) is a significant health burden worldwide, with at least 300,000 cases reported in the United States [1]. In Korea, the number of SCA cases has increased 1.5-fold, from 19,480 in 2006 to 29,832 in 2016 [2]. Despite an overall decline in cardiovascular morbidity and mortality over the past few decades, the survival rate after resuscitated SCA remains low, ranging from 3% to 20% across different communities [3]. Although the rates of survival and neurological recovery in Korea have also increased each year, with the former rising 3.3-fold from 2.3% in 2006 to 7.6% in 2016, they remain low [2]. Therefore, primary prevention is crucial to reducing SCA-related morbidity and mortality [4].

The risk factors for SCA have been extensively studied at various levels. Factors such as age, gender, medical history, and lifestyle behaviors—including smoking, physical activity, and diet—have all been identified as contributing to the risk of SCA [4,5]. Based on this knowledge, cardiovascular risk factors, such as hypertension, diabetes, and cardiac diseases, as well as lifestyle modifications like a healthy diet, regular exercise, and quitting smoking, have been recommended. Moreover, recent studies have focused on the association between socioeconomic status, occupation type, and SCA [6,7,8]. The incidence of SCA at home or in a residential institution was higher in poorer neighborhoods of American and Canadian sites studied [9]. In Korea, community-level socioeconomic status has been associated with resuscitation, survival to discharge, and neurologic outcomes [10,11,12].

Efforts to identify the relationship between various occupational characteristics and diseases, as well as mortality, and to prevent these outcomes have been carried out in various studies [13,14]. There is a long-standing interest in the impact of job strain on health outcomes because different job types may exert varying degrees of physical and psychological stress on the body. Occupational status can influence psychosocial factors related to SCA, such as job-related physical activity, environment, responsibility, and stress [8]. In office settings, mental stress also appears to precipitate SCA, possibly by directly impacting the cardiac ion channels that control the heart’s electrical properties, which may lead to ventricular fibrillation, the arrhythmia underlying SCA [15]. Additionally, work schedules and patterns can have distinct physiological and psychological effects. One study reported high-stress life events and social strain to be associated with a higher risk of coronary heart disease (CHD), and job strain also interacted with social strain in relation to CHD risk [16]. Other research has shown that working environments can significantly elevate the risk of disease, including CHD and stroke [17,18,19].

To our knowledge, no studies have explored the association between SCA and occupational environments, including job type, working hours, and duty type. We compared healthy adults and SCA patients to evaluate the relationship between employment environment and SCA.

## 2. Materials and Methods

### 2.1. Study Design and Setting

This was a case–control study conducted as part of the CAPTURES (Cardiac Arrest Pursuit Trial with Unique Registration and Epidemiologic Surveillance) project in Korea. The CAPTURES project was a prospective multicenter initiative aimed at identifying risk factors for SCA and developing preventive strategies for affected patients [4]. The project involved the development of a hospital-based registry and included 17 emergency departments (EDs) in Korea. This study, initiated in 2018, used a prospective case–control design. Data for the case group were collected through structured interviews conducted by investigators at hospital emergency centers. Control group data were obtained from individuals visiting public health centers for routine medical checkups, with data collection performed through individual interviews.

The study was approved by the ethics committees of all participating institutions. In compliance with the Declaration of Helsinki, all participants (or their proxies) provided written informed consent prior to their participation in the study [20]. This project is registered at ClinicalTrials.gov (NCT 03700203).

### 2.2. Study Sample

Seventeen EDs participated in data collection from patients aged 20–79 years with SCA resulting from medical causes. Individuals younger than 20 years were excluded because they are not typically employed. Ineligible patients included those with cardiac arrest due to trauma, drowning, poisoning, burns, asphyxia, or hanging, as well as those who did not provide informed consent. The project also excluded patients with the following conditions: terminal illnesses, those currently under hospice care, pregnant individuals, individuals living alone or experiencing homelessness, those lacking a reliable source of information, or individuals in possession of a “Do Not Resuscitate” card [4]. When SCA patients visited the emergency room, regardless of survival, researchers conducted structured surveys through direct interviews after obtaining consent from the patient for them to participate in the study. If the patient was deceased or unconscious, the interview was conducted with a direct family member.

Community-based controls were recruited from nine centers, representing both metropolitan and non-metropolitan areas. All voluntary controls were enlisted at collaborating public health centers or various community centers where the project was actively promoted [4]. The control group comprised individuals who visited for health checkups, and surveys were conducted with those who agreed to participate after confirming their willingness. All volunteers completed the structured questionnaires independently.

### 2.3. Data Collection

Emergency physicians at the participating EDs gathered information through structured surveys conducted during face-to-face interviews with patients or their families, depending on the patient’s ability to communicate or the severity of their condition. Structured questionnaires were used, and both cases and controls underwent physical examinations, including blood sampling. Information on demographic factors, comorbidities, socioeconomic status, and occupational factors was systematically collected.

The structured questionnaires used in this project covered general information, ED interviews, ED management, cardiac panels, prehospital management, hospital care, and prognosis. The ED interview items included in this study were family medical history, past medical history, quality of life, sleep patterns, eating habits, vaccination history, education, marital status, and economic and social status, as well as employment type, occupation, working hours, and work patterns. The questionnaires for the control group were identical to those for the case group, except for questions related to medical treatment.

Occupation was categorized as either employer or employee. Employers were further classified as sole proprietors or business owners. Employees were categorized as office workers, professional workers, service workers, or laborers. Unemployed individuals included students, homemakers, and job seekers.

Socioeconomic status was assessed based on individuals’ insurance coverage. Unlike national health insurance, car insurance, and travel insurance, those receiving medical aid services or foreign workers without health insurance were defined as being in a low-economic state.

Occupational factors included working hours and work patterns. Working hours were evaluated in terms of daily working hours, mean weekly working days, and mean weekly working hours. Work patterns involved evening, night, Saturday, and Sunday shifts, as well as instances of working over 10 h in a day.

All data were transferred to the data quality management committee, where quality-control checks and statistical analyses were performed. The committee provided feedback to each center coordinator on data quality management during the monthly meetings [4].

### 2.4. Statistical Analyses

To minimize bias from confounding variables, we implemented propensity score matching between the case and control groups. The matching variables included age, gender, and socioeconomic status, and the matching process was performed on a one-to-one basis. Socioeconomic status was divided into the national health insurance group and the medical aid group.

Continuous data are expressed as mean (standard deviation, SD) or median (interquartile range, IQR), as appropriate. Categorical data are presented as numbers with corresponding percentages. For comparisons, Student’s *t*-test and the Mann–Whitney U-test were employed for continuous variables, while chi-square or Fisher’s exact tests were used for categorical variables. Odds ratios (OR) and 95% confidence intervals (CI) were calculated using logistic regression analysis.

A two-tailed *p*-value < 0.01 was considered statistically significant. All analyses were performed using R version 3.6.3 (R Foundation for Statistical Computing, Vienna, Austria) and IBM SPSS Statistics for Windows, version 20.0 (IBM Corp., Armonk, NY, USA).

## 3. Results

### 3.1. Study Sample and Characteristics

**Figure 1:**
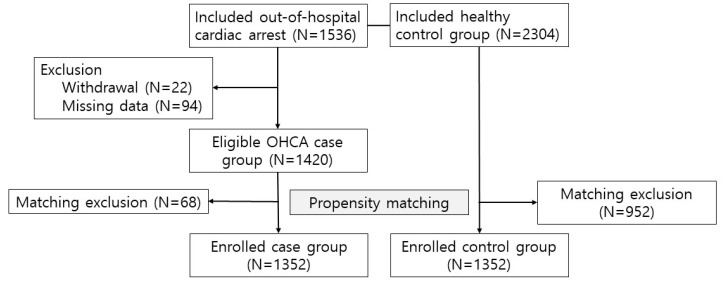
Inclusion and exclusion flow chart.

This study was conducted from September 2017 through December 2022. During this period, 1536 SCA patients and 2304 healthy controls were initially enrolled. After excluding 22 cases due to withdrawal from the study and 94 cases with missing data, a total of 1420 patients were enrolled. Subsequently, propensity score matching was employed, resulting in 1352 individuals in each group for further analysis (Figure 1). The mean ages of the cases and controls were 60.7 ± 12.6 and 60.1 ± 12.4, respectively. Males comprised 75.3% of individuals in both groups. Additionally, 5.4% of each group was classified as having a low economic status.

The SCA case group had a higher prevalence of comorbidities compared to the healthy control group. These comorbidities included hypertension (47.1% vs. 39.1%, *p* < 0.01), diabetes (30.0% vs. 15.8%, *p* < 0.01), dyslipidemia (15.2% vs. 25.4%, *p* < 0.01), coronary artery disease (7.7% vs. 0.2%, *p* < 0.01), stroke (9.3% vs. 3.5%, *p* < 0.01), chronic kidney disease (8.7% vs. 0.5%, *p* < 0.01), and malignancy (8.6% vs. 5.8%, *p* = 0.02). Conversely, obesity was less prevalent in the SCA case group (3.9% vs. 11.8%, *p* < 0.01) (Table 1).

### 3.2. Occupational Distribution

Of all 2704 enrolled participants, 1445 (53.4%) were employed and 1259 (46.6%) were unemployed. Among the 449 employers, the rate of sole proprietorship did not differ significantly between the two groups (20.6% vs. 22.5%, *p* = 0.39). However, the proportion of business owners was higher in the SCA case group compared to the healthy control group (13.5% vs. 6.5%, *p* < 0.01). Among employees, the proportion of professional workers was lower in the SCA case group compared to the healthy control group (23.6% vs. 31.6%, *p* < 0.01). In contrast, labor workers were more prevalent in the SCA case group (27.7% vs. 17.8%, *p* < 0.01). There were no significant differences in the proportions of officers (23.4% vs. 24.3%, *p* = 0.70) and service workers (25.1% vs. 26.1%, *p* = 0.66) between the two groups. Among the unemployed individuals, the SCA case group had a significantly higher proportion of job seekers compared to the healthy control group (22.3% vs. 4.2%, *p* < 0.01), while retirees were more prevalent in the healthy control group (18.5% vs. 37.9%, *p* < 0.01) (Table 2).

### 3.3. Working Times and Patterns

**Figure 2:**
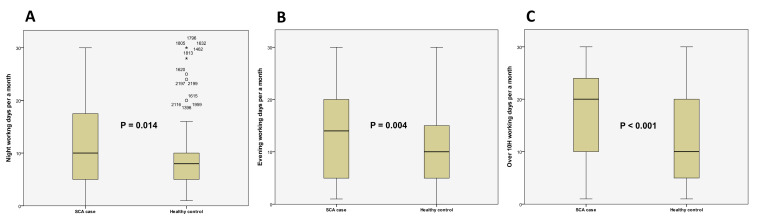
Comparison of night/evening shifts and days with over 10 h of work between cases and controls. (**A**) Number of night shifts per month in the SCA case and healthy control groups. An asterisk (*) denotes an outlier, which is an observation that falls outside the outer fence of the box plot. And a circle (○) denotes an anomaly, which is an observation that falls outside the inner fence of the box plot. (**B**) Number of evening shifts per month in the SCA case and healthy control groups. (**C**) Number of days with over 10 h of work per month in the SCA case and healthy control groups. In all analyses, the SCA case group showed a significantly higher frequency, with *p*-values of 0.014, 0.004, and <0.001, respectively. SCA: sudden cardiac arrest.

Individuals experiencing SCA had shorter overall working times than the healthy control group. In the SCA group, the mean daily working hours were 2.7% longer, and weekly working hours were 0.3% longer (8.60 ± 2.85 h vs. 8.37 ± 2.67 h, *p* = 0.14; 44.00 ± 19.77 h vs. 43.86 ± 15.61 h, *p* = 0.89, respectively). Saturday and Sunday duties were more frequent in the SCA group, although the difference was not significant (OR 1.07, 95% CI 0.99–1.15; OR 1.02, 95% CI 0.95–1.10, respectively). However, the SCA group had a higher frequency of evening and night shifts per month. Evening shifts were 4% more common, and night shifts were 5% more frequent in the SCA group (13.77 ± 8.91 days vs. 11.19 ± 8.19 days, *p* < 0.01; 11.40 ± 7.75 days vs. 9.14 ± 6.31 days, *p* = 0.01, respectively). Additionally, long working days (>10 h/day) were 6% more frequent in the SCA group (17.5 ± 8.33 days/month vs. 12.86 ± 9.37 days/month, *p* < 0.01) (Table 3, Figure 2).

## 4. Discussion

The type of work and work intensity are factors that have a significant impact on health. Many previous studies have shown that the risk of cardiovascular and cerebrovascular diseases increases as work intensity becomes more severe. In this case–control study, the healthy control group had a higher prevalence of current employment and worked more days and hours. However, prolonged single-shift work, as well as evening and night shifts, were more common in the out-of-hospital cardiac arrest group. Interestingly, although the amount of work in a person’s life might not correlate with mortality, excessive labor was associated with an increased risk.

### 4.1. Occupation Type and SCA

One previous case–control study examined the association between occupation type and SCA. The authors classified occupations into white-collar, blue-collar, and homemaker. White-collar jobs included non-manual work, such as professional, administrative, managerial, and higher technical roles, as well as office and sales/service positions. Blue-collar jobs encompassed construction, production, maintenance, transportation, and other labor-intensive work. They reported a 1.67-fold higher risk of SCA events (95% CI 1.26–2.23; *p* < 0.001) in male white-collar workers compared to blue-collar workers [8]. In our study, we conducted a detailed investigation into various occupations, categorizing individuals as employers, employees, and unemployed. The employer group comprised sole proprietors and business owners; the employee group included professionals, service workers, and laborers; and the unemployed category consisted of job seekers and retirees. We categorized individuals by occupation because we believed that the impact of mental stress and labor intensity would vary depending on job position. In the employer group, unlike sole proprietors, business owners who had multiple employees were more likely to be in the SCA case group. This may be due to the stress associated with managing a larger business and having a high decision-making burden.

### 4.2. Psychological Stress and SCA

A previous study reported that a high decision-making burden significantly increases the risk of CHD, especially among women (adjusted relative risk [RR] 2.80, 95% CI 1.09–7.17) [21]. Psychological distress has been identified as a risk factor for coronary and cerebrovascular events and related mortality [22,23,24,25]. Among employees, while professional workers who perform more specialized tasks were significantly fewer in the SCA case group, we found that laborers who engage in physical work were more likely to be in the SCA group. This finding differs from previous studies, as not all physical work leads to a healthy lifestyle. In fact, the risk of accidents can increase, and repetitive physical tasks can cause various musculoskeletal diseases. Eaker et al. also reported that operators and laborers have twice the mortality rate, especially among men (adjusted RR 2.02, 95% CI 1.24–3.29) [21]. The working environment is also an important factor [26]. Among the unemployed, contrary to expectations, job seekers were more likely to be in the SCA case group, while retirees were more likely to be in the healthy control group. This may be due to selection bias, as more retirees than job seekers undergo regular health checkups.

### 4.3. Working Hours and SCA

One of the most significant job strains is working hours. Previous studies have reported that long working hours increase the risk of cardiovascular and cerebrovascular diseases. Work-related cerebrovascular disease, in particular, was more strongly associated with long working hours than cardiovascular disease. A meta-analysis reported that individuals who work 55 h or more per week have a 1.3 times higher risk of incident stroke compared to those working standard hours [18]. Another meta-analysis found that, compared with working 35–40 h per week, working 55 h or more per week may lead to a significant increase in the risk of dying from ischemic heart disease (RR 1.17, 95% CI 1.05–1.31) [27]. In Korea, weekly working hours exceeding 60.75 h are associated with work-related cerebrovascular disease with high specificity (94%) [28]. Consequently, the Labor Standards Act in Korea limits weekly working hours to 52 h. However, in this study, we could not analyze the impact of extreme working hours on SCA because our population’s mean weekly working hours did not exceed 45 h.

On the other hand, when evaluating daily prolonged work hours, workdays exceeding 10 h were 6% more frequent in the SCA group, which was statistically significant. Although many studies have been conducted on the risks of prolonged working hours, most analyses have focused on mean weekly working hours. To our knowledge, no studies have investigated the impact of temporary episodes of extended working hours (over 10 h) on health. Sudden death from overwork is often caused by cardiovascular disease and is believed to result from a repetitive triggering of the stress response [29]. Moreover, behavioral mechanisms, such as physical inactivity, might also link long working hours to stroke, as supported by evidence of an increased risk of incident stroke in individuals who sit for extended periods at work [30]. Therefore, prolonged overwork in the office may increase the risk of SCA. Through this study, we found that even if the weekly working hours are not long, the risk of cardiac arrest increases with a higher number of days involving long working hours. Therefore, to maintain health, it is advisable to avoid long overtime work whenever possible and to minimize the number of overtime workdays.

### 4.4. Night Shift Working Patterns and SCA

Human beings have physiological systems that are rhythmically timed to synchronize enzyme, hormone, and energy levels with organ activity [31]. A biological rhythm period is the time needed to complete one full cycle of a rhythm, which is approximately 24 h and is known as the circadian rhythm. The circadian rhythm influences heart rate, blood pressure, and serum levels of epinephrine and cortisol concentrations [32]. When people engage in rotating or night shift work, their circadian rhythms cannot quickly adapt, leading to desynchronization of many physiological systems [33]. Wang et al. reported a significantly increasing trend in the risk of incident atrial fibrillation from “day workers” to “shift workers who never/rarely work night shifts”, to “those with some night shifts”, and finally to “those with usual/permanent night shifts”. The highest risk was associated with usual or permanent night shifts (hazard ratio 1.16, 95% CI 1.02–1.32) [34]. Long-term exposure to night shifts—working night shifts over 3–8 times per month—is associated with an increased risk of atrial fibrillation. Additionally, individuals who regularly or permanently work night shifts have a significantly higher risk of developing CHD [34]. In the present study, we showed that individuals with SCA also have a significantly higher frequency of night shifts. Moreover, the SCA case group had more evening shifts. On the other hand, working daytime shifts on Saturdays or Sundays did not affect the incidence of SCA.

### 4.5. Limitations

There were some limitations to this study, including that this was a case–control study rather than an interventional study, meaning that potential biases could not be fully controlled. Specifically, since data for the control group were collected from a health screening center, the study may have included data from more health-conscious individuals with more time and economic resources. Nevertheless, we controlled for socioeconomic status through propensity matching to minimize social differences between the groups. In the SCA case group, patient information was mostly obtained through caregivers because the patients were unresponsive or incapacitated immediately after experiencing cardiac arrest and could not provide information themselves. This may limit the accuracy of the data but is an inherent limitation of case–control studies with SCA as the endpoint. Another limitation is that we measured risk factors through a survey, which might have been associated with misclassification and recall bias. However, our protocol included specific questions that the interviewer should ask when conducting the survey. Unfortunately, although the data collection questionnaire included questions about comorbidities, it lacked detailed information about the patients’ medications, preventing us from assessing the effects of their usual treatments. Another limitation was that although the participating hospitals—which enrolled the SCA cases—were evenly distributed across the country, they were primarily located in areas with high population densities, as they were university hospitals. This distribution may not be representative of rural populations. Furthermore, the case and control groups were recruited from different regions, which could introduce additional bias.

## 5. Conclusions

We investigated occupational factors that may influence SCA and found an increased risk of SCA associated with both blue-collar work and business ownership. Additionally, the risk of cardiac arrest appears to increase in workers who frequently work evening and night shifts, as well as those who consistently work overtime, exceeding 10 h a day. To overcome the limitations of our study, further well-designed studies, such as nationwide data analyses or cohort studies, are required.

## Figures and Tables

**Table 1:** Propensity-score-matched variables and comorbidities.

Variables	SCA Case Group (n = 1352)	Healthy Control Group (n = 1352)	*p* Value
Matching variables			
Age (years)	60.7 ± 12.6	60.1 ± 12.4	0.22
Sex (male)	1018 (75.3)	1018 (75.3)	>0.99
Economic status (low)	73 (5.4)	73 (5.4)	>0.99
Comorbidities			
Hypertension	637 (47.1)	529 (39.1)	<0.01
Diabetes	406 (30.0)	214 (15.8)	<0.01
Dyslipidemia	205 (15.2)	343 (25.4)	<0.01
Obesity	53 (3.9)	159 (11.8)	<0.01
Coronary artery disease	100 (7.7)	3 (0.2)	<0.01
Stroke	126 (9.3)	47 (3.5)	<0.01
Chronic kidney disease	117 (8.7)	7 (0.5)	<0.01
Malignancy	116 (8.6)	78 (5.8)	0.02

SCA: sudden cardiac arrest.

**Table 2:** Occupational distribution between sudden cardiac arrest and healthy control groups.

Variables	SCA Case Group (n = 1352)	Healthy Control Group (n = 1352)	*p* Value
Employer			
Sole proprietor	131 (20.6)	180 (22.5)	0.39
Business owner	86 (13.5)	52 (6.5)	<0.01
Employee			
Officer	143 (23.4)	191 (24.3)	0.70
Professional/technical worker	144 (23.6)	248 (31.6)	<0.01
Service worker	153 (25.1)	205 (26.1)	0.66
Blue collar labor	169 (27.7)	140 (17.8)	<0.01
Unemployed people			
Job seeker	158 (22.3)	23 (4.2)	<0.01
Retirement	131 (18.5)	208 (37.9)	<0.01

SCA: sudden cardiac arrest.

**Table 3:** Working hours and patterns associated with sudden cardiac arrest in the SCA group.

Logistic Regression Analysis	SCA Case Group (n = 1352)	Healthy Control Group (n = 1352)	Univariate OR (95% CI)
Working times			
Mean daily working time (hours)	8.60 ± 2.85	8.37 ± 2.67	1.03 (0.99–1.07)
Mean weekly working day (days)	5.08 ± 0.99	5.24 ± 0.98	0.85 (0.76–0.96)
Mean weekly working time (hours)	44.00 ± 19.77	43.86 ± 15.61	1.00 (0.99–1.01)
Working patterns (days)			
Monthly evening working day	13.77 ± 8.91	11.19 ± 8.19	1.04 (1.01–1.06)
Monthly night working day	11.40 ± 7.75	9.14 ± 6.31	1.05 (1.01–1.09)
Monthly Saturday working day	3.38 ± 1.72	2.97 ± 2.63	1.07 (0.99–1.15)
Monthly Sunday working day	3.04 ± 1.68	2.91 ± 2.86	1.02 (0.95–1.10)
Monthly over 10 h working day	17.5 ± 8.33	12.86 ± 9.37	1.06 (1.03–1.08)

SCA: sudden cardiac arrest; OR: odds ratio; CI: confidence interval.

## Data Availability

The research data is not available due to the lack of permission to provide personal information.
